# Treadmill Training for Common Marmoset to Strengthen Corticospinal Connections After Thoracic Contusion Spinal Cord Injury

**DOI:** 10.3389/fncel.2022.858562

**Published:** 2022-04-22

**Authors:** Takahiro Kondo, Risa Saito, Yuta Sato, Kenta Sato, Akito Uchida, Kimika Yoshino-Saito, Munehisa Shinozaki, Syoichi Tashiro, Narihito Nagoshi, Masaya Nakamura, Junichi Ushiba, Hideyuki Okano

**Affiliations:** ^1^Department of Physiology, Keio University School of Medicine, Tokyo, Japan; ^2^Graduate School of Science and Technology, Keio University, Yokohama, Japan; ^3^Department of Rehabilitation Medicine, Keio University School of Medicine, Tokyo, Japan; ^4^Department of Orthopaedic Surgery, Keio University School of Medicine, Tokyo, Japan; ^5^Department of Biosciences and Informatics, Faculty of Science and Technology, Keio University, Yokohama, Japan

**Keywords:** marmoset, spinal cord injury, rehabilitation, kinematics, locomotion, treadmill training

## Abstract

Spinal cord injury (SCI) leads to locomotor dysfunction. Locomotor rehabilitation promotes the recovery of stepping ability in lower mammals, but it has limited efficacy in humans with a severe SCI. To explain this discrepancy between different species, a nonhuman primate rehabilitation model with a severe SCI would be useful. In this study, we developed a rehabilitation model of paraplegia caused by a severe traumatic SCI in a nonhuman primate, common marmoset (*Callithrix jacchus*). The locomotor rating scale for marmosets was developed to accurately assess the recovery of locomotor functions in marmosets. All animals showed flaccid paralysis of the hindlimb after a thoracic contusive SCI, but the trained group showed significant locomotor recovery. Kinematic analysis revealed significantly improved hindlimb stepping patterns in trained marmosets. Furthermore, intracortical microstimulation (ICMS) of the motor cortex evoked the hindlimb muscles in the trained group, suggesting the reconnection between supraspinal input and the lumbosacral network. Because rehabilitation may be combined with regenerative interventions such as medicine or cell therapy, this primate model can be used as a preclinical test of therapies that can be used in human clinical trials.

## Introduction

A thoracic spinal cord injury (SCI) disrupts the connectivity between the brain and lumbar spinal circuits and leads to lower limb paralysis. Rehabilitative approaches are being explored to improve locomotor function, which is an important factor affecting the quality of life after SCI. Mechanisms of rehabilitative effects on locomotor recovery have been gradually studied over the past decade (Fouad and Tetzlaff, 2012; Shinozaki et al., 2016). These mechanisms include the upregulation of neurotrophic factors (Vaynman and Gomez-Pinilla, 2005; Tashiro et al., 2015), an increase in the sprouting and regeneration of neural fibers (Girgis et al., 2007), and the facilitation of cortical map changes (Ishida et al., 2016). Rehabilitation has been shown to improve the recovery of stepping movements even after a severe SCI in various animal models, including mice (Fong et al., 2005), rats (Cha et al., 2007), and cats (Leon et al., 1998). However, in patients with a severe SCI (A or B on the American Spinal Injury Association Impairment Scale [AIS]), intensive treadmill training improved electromyographic activity but not overground walking (Dietz et al., 2002).

The discrepancy in rehabilitation results between human and animal models may be due to differences in walking recovery mechanisms between these two species. Although the central nervous system (CNS) of rodents is small, their spinal cord accounts for 30% of their net CNS weight, whereas that of humans is only 3% (Swanson, 1995). The recovery of walking after incomplete lesions in humans depends on a descending input from the motor cortex and the ability to strengthen corticospinal connections (Yang and Gorassini, 2006), and the recovery of walking function in patients with SCI seems to be highly dependent on supraspinal input (Cote et al., 2017). For example, the firing of primary motor cortex (M1) neurons is not modulated during feline walking (Drew et al., 2002), whereas cortical EEG signals are modulated during human walking (Petersen et al., 2012). Other studies have shown that stimulation of the corticospinal tract (CST) improves walking in patients with a chronic incomplete SCI (Benito et al., 2012). These reports suggest that walking function by the human spinal cord is highly dependent on cortical commands. Inconsistent results in lower mammals and primates/humans suggest that conducting studies on nonhuman primates is beneficial to bridge this knowledge gap.

Previous studies on nonhuman primates have shown the importance of the motor cortex in locomotion (Rosenzweig et al., 2010; Friedli et al., 2015; Capogrosso et al., 2016) but have created a hemisection model that is not clinically relevant. On the other hand, several studies have been conducted in common marmosets with a contusive SCI to facilitate the successful progress of potential treatments to clinical trials (Iwanami et al., 2005; Kitamura et al., 2011; Kobayashi et al., 2012; Iwai et al., 2015). We tested whether locomotor rehabilitation could lead to a regain of supraspinal input and improved walking recovery after a contusive SCI. To answer this question, we developed a thoracic contusive SCI model of nonhuman primates and investigated their kinematics, electrophysiological, and histological analyses.

## Materials and Methods

### Animals

In the present study, nine common marmosets (*Callithrix jacchus*; female, bodyweight: 280–350 g, age: 2–5 years) were used. All animal experiments were approved by the Animal Research Committee of Keio University School of Medicine (approval number: 11,006) and conformed to the National Institutes of Health (1996) guidelines.

Each marmoset was assigned to one of the two groups: trained (*n* = 3, Marmoset *T*_K_, *T*_M_, and *T*_D_) and untrained (*n* = 3, Marmoset UT_R_, UT_W_, and UT_G_). For scale development and validation of the open field scoring test, UT_Y_ and UT_H_ underwent 175 kdyn, and UT_I_ underwent a 200-kdyn contusive SCI.

### Model of SCI

Surgery was performed under general anesthesia induced by intramuscular injection of ketamine (30 mg/kg, Daiichi-Sankyo, Tokyo, Japan), xylazine (2.5 mg/kg, Bayer Healthcare, Monheim, Germany), and atropine sulfate (0.05 mg/kg, Mitsubishi Tanabe Pharma, Osaka, Japan), and was maintained by 1–1.5% isoflurane (MSD, Tokyo, Japan). Pulse and arterial oxygen saturation were monitored during surgical procedures. After laminectomy at the T10 level, a 250-kdyn (UT_Y_ and UT_H_ underwent 175 kdyn, and UT_I_ did 200 kdyn) contusive SCI was inflicted on the exposed dura mater (without durotomy) using a commercially available SCI device (IH Impactor, Precision Systems and Instrumentation, KY, USA).

To maintain a desirable state of health after SCI, intensive daily care was done. To avoid constipation, liquid food or milk was served instead of solid food from 2 days pre-injury to 5 days after injury. Manual bladder emptying was performed two times a day. One week after injury, ceftriaxone (30 mg/kg, Nichi-Iko Pharmaceutical Co., Ltd., Tokyo, Japan) and butorphanol tartrate (5.0 mg/kg, Meiji Seika, Tokyo, Japan) were injected intramuscularly, and saline was administered subcutaneously. Paralyzed animals were given adequate quantities of food and water until they recovered their ability to ingest food and water without assistance. Marmosets regained their bowel, bladder, and autonomic function 1 week after SCI.

### Treadmill Training

**Figure 1:**
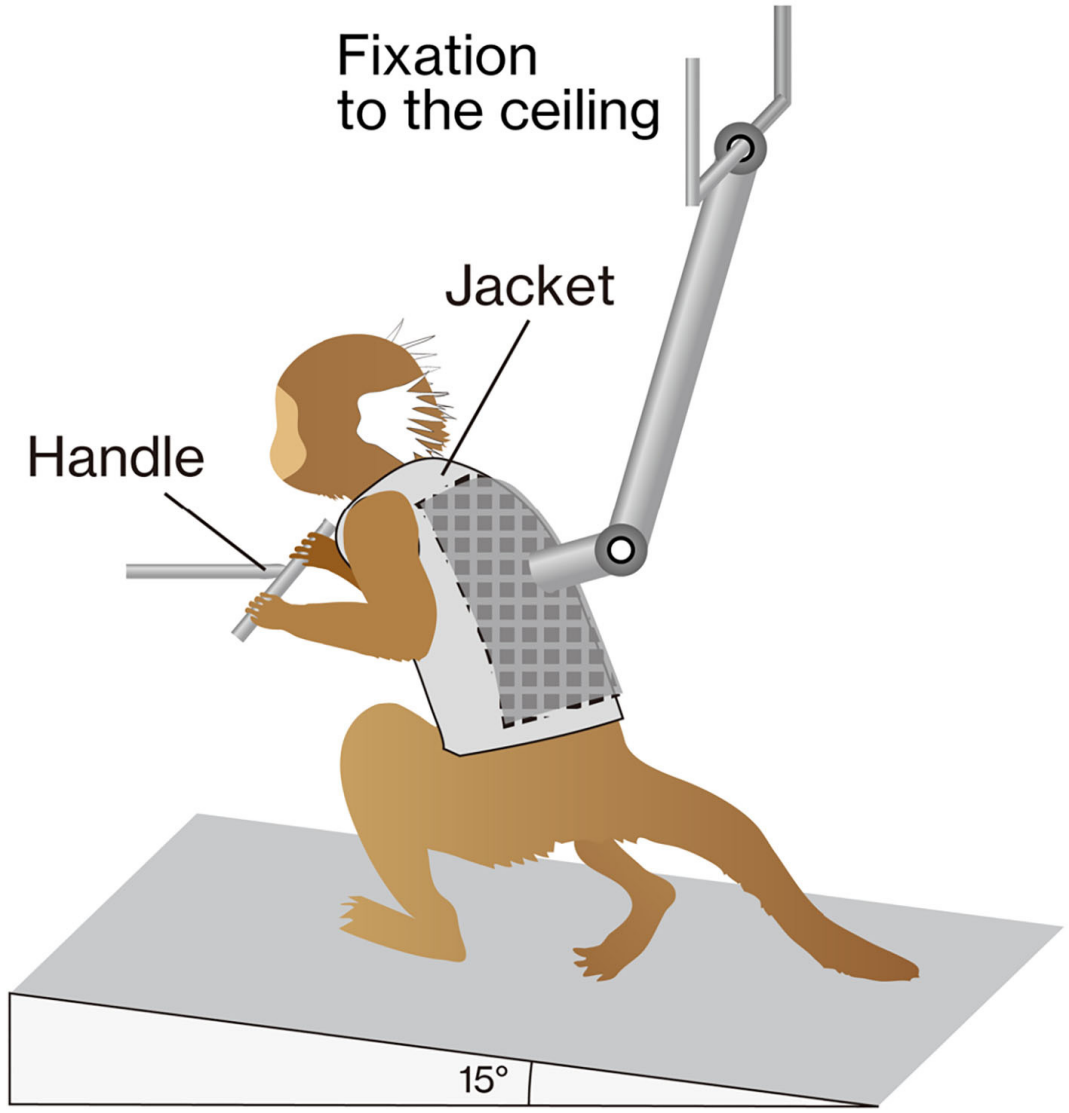
The training apparatus. The marmoset was placed on a treadmill (inclined by 15°) with a custom-made jacket attached to the ceiling for weight support. The gripping handle was positioned in front of the animal. During training, marmosets were rewarded to keep them stable.

Animals were acclimatized for 1–2 weeks, so that they would remain calm when walking on a custom-made treadmill device for partial body weight-supported bipedal treadmill walking (Figure 1). Prior to intervention, animals were habituated to wear a custom-made jacket with a Velcro® strip (Velcro, London, UK), which was used to attach the marmoset to a weight support and to grip the bar in front of them. The treadmill device was inclined at 15°, and the speed of the treadmill was set to 10.0 cm/s. We know empirically that marmosets that were able to move ankle joints 2 weeks after injury would subsequently regain locomotor functions without rehabilitation. Hence, to ensure that the contusive injury was correctly formed, rehabilitation was initiated after confirming little or no ankle movement 2 weeks after injury. Rehabilitation sessions were performed 5 days/weeks for 3 consecutive weeks (2–4 weeks after injury) and for 30 min with manual assistance. During the stance phase, the experimenter helped the animals swing by holding their ankles and carefully pressing their plantars firmly against the floor.

### Marmoset Motor Scale

All animals showed paraplegia after injury, and their locomotor functions gradually recovered. Because reliable outcome measures are required to assess the recovery of locomotor functions in marmosets, we developed original scoring scales based on the previous locomotion scale in rodents (BBB; Basso et al., 1995) and mice (BMS; Basso et al., 2006). This scale was named Marmoset Motor Scale for Locomotion (MMS). Table 1 describes how the motor characteristics of marmosets differ from those of rodents when assessing the recovery of marmoset motor function. Because marmosets move faster than rodents and have a relatively long moving span, evaluation requires video recording. In rats, toe clearance was assessed based on auditory cues with their short leg lengths, whereas in marmosets, it was visually assessed due to their larger size and longer legs. We used a large, open field enclosed in a plexiglass sidewall (0.3 m sidewall height and a 4 × 2 m rectangle).

**Table 1:** Characteristics of locomotion in the open field.

	**Mouse**	**Rat**	**Marmoset**
Moving Speed	Relatively rapid	Slow	Rapid
Moving span	Short bouts	Relatively long	Long
Recovery of FL-HL coordination	Simultaneously recover with paw position	Precede recovery of paw position	Precede recovery of paw position
Leg length	Short	Relatively long	Long
Toe clearance	Hard to assess	Assessed from auditory cues	Assessed visually

### Kinematics Analysis

All animals walking at a self-speed were recorded before the injury and at 2, 5, 8, and 11 weeks after injury (Figure 1). All of the procedures used have been previously described in detail (Shimada et al., 2017). Briefly, a clear acrylic walkway (1,350 mm in length, 90 mm in width, and 180 mm in height) with a black rubber floor sheet was used to avoid slipping while walking. Animals were habituated to the walkway before recording to ensure their stability while walking straight on it. The walking of the animals was recorded using two cameras (MEMRECAM GX-3; NAC Imaging Technology, Tokyo, Japan), which were synchronized through an internal trigger (150 or 200 Hz). Before each recording, a 4-mm reflective marker was attached bilaterally to the shaved skin, overlying the following specific bony landmarks: acromion of the scapula (shoulder), greater trochanter of the femur (hip), femoral condyle (knee), lateral malleolus of the fibula (ankle), and the fifth metatarsal head (MTP).

Each marker was tracked from each video, and *x*- and *y*-coordinates were automatically quantified using the KinemaTracer software (Kissei Comtec Co., Ltd., Nagano, Japan). The walking cycle was defined as one foot contact to the following foot contact and was subdivided into the stance and swing phase by foot-lift. Each foot contact was visually determined and fed into the KinemaTracer software.

The body was modeled as an interconnected chain of rigid segments and was visualized as stick pictures. In addition, overlaying the trajectory of the MTP in the swing phase for each trial made it easier to visually compare the changes in hindlimb movements.

Each joint angle was defined as the narrower angle between the two segments across each joint. The amplitude of angular joint movements was measured as the difference between the maximum positions in flexion and extension. To quantify the similarity of joint angles before and after injury, the cross-correlation was applied to the time-normalized waveform, and the highest correlation coefficient determined the similarity. Furthermore, based on previous studies (Takeoka et al., 2014; DiGiovanna et al., 2016; Sato et al., 2021), 47 kinematic parameters were extracted (Table 2) and principal component analysis (PCA) was applied for a comprehensive evaluation of changes. Note that UT_G_ was not included in the kinematics analysis because it was not possible to extract gait parameters because the hindlimb of UT_G_ was dragged until 11 weeks after SCI. Therefore, the kinematics data of the untrained group only included the other two marmosets.

**Table 2:** Kinematic parameters used for principal component analysis (PCA).

**Temporal features of gait**	**Joint angle and segmental oscillation**	
Cycle duration (s)	Maximal angle (deg.)	Thigh
Stance duration (s)		Shank
Swing duration (s)		Foot
Relative stance duration, %		Hip
		Knee
**Endpoint trajectory**		Ankle
Stride length (cm)	Minimal angle (deg.)	Thigh
Step length (cm)		Shank
Maximal forward position of foot (cm)		Foot
Maximal backward position of foot (cm)		Hip
Maximal forward position of foot (x) (cm)		Knee
Maximal backward position of foot (x) (cm)		Ankle
Step height (cm)	Angle Amplitude (deg.)	Thigh
Maximal speed during swing (cm/s)		Shank
Time of maximal velocity during swing, %		Foot
Acceleration at swing onset (cm/s^∧^2)		Hip
Endpoint velocity (x) (cm/s)		Knee
		Ankle
**Lags with maximum cross correlation** **between segments**	Maximal angle velocity (deg./s)	Hip
		Knee
Thigh vs Shank		Ankle
Shank vs Foot	Minimal angle velocity (deg./s)	Hip
Hip vs Knee		Knee
Knee vs Ankle		Ankle
Ankle vs Foot	Angle velocity amplitude (deg./s)	Hip
		Knee
		Ankle

### Intracortical Microstimulation

Before the injury, the head pipe was implanted in a manner similar to that previously described (Kondo et al., 2018). Marmosets were anesthetized by an intramuscular injection of ketamine (30 mg/kg) and xylazine (2.5 mg/kg). Body temperature and oxygen saturation levels were monitored. Anesthetized animals were mounted on a custom-made stereotaxic apparatus (IMPACT-1000B; Muromachi Kikai, Tokyo, Japan), the skull was exposed, and two polyetheretherketone (PEEK) pipes (Muromachi Kikai, Japan) were attached to the skull with dental cement (UNIFAST II, GC, Japan). Two pipes were parallelly placed over the frontal and occipital areas, and both pipes were flanked by small stainless steel bars.

The next day, marmosets fitted with head pipes were sedated with an intramuscular injection of ketamine (30 mg/kg), atropine sulfate (0.05 mg/kg), ceftriaxone (30 mg/kg), and dexamethasone (0.30 mg/kg, MSD, Tokyo, Japan), and placed in a custom-made stereotaxic chair (Muromachi Kikai, Tokyo, Japan). The abovementioned two pipes were used for head fixation; this allowed us to avoid the use of painful tooth/eye and ear bars. Therefore, marmosets received intracortical microstimulation (ICMS) under natural conditions without xylazine treatment. A large craniotomy (7 × 7 mm square) was performed in the area of the right motor cortex, and tungsten electrodes were implanted to deliver ICMS (10 biphasic pulses composed of 0.2-ms cathode and 0.2-ms anode pulses at 333 Hz). The locations of the hindlimb and forelimb M1 areas were estimated based on the observation of evoked motor responses. At the end of ICMS, artificial dura (5 × 5 mm) (PPX-03060; Gore, Tokyo, Japan) was applied to the brain surface, and a Kwik-Sill® silicone elastomeric adhesive (World Precision Instruments, FL, USA) was mounted.

### Histological Analysis

At 11 weeks after injury, marmosets were deeply anesthetized with an overdose of sodium pentobarbital (50 mg/kg; Kyoritsu Seiyaku, Tokyo, Japan) and intracardiac perfusion of 0.1 M potassium phosphate-buffered saline (PBS; pH 7.3), followed by 4% paraformaldehyde (PFA; Merck, NY, USA) in 0.1 M PBS. The spinal cords were removed, post-fixed overnight in 4% PFA in 0.1 M PBS, soaked overnight in 10% sucrose, followed by 30% sucrose, and then cut serially into 50-μm thick coronal sections using a freezing microtome (Retoratome, REM-710; Yamato, Saitama, Japan). The sections located at the lesion epicenter were stained with Luxol fast blue (LFB) to evaluate the myelinated area after injury. For NF-H immunohistochemistry, the sections located 500 μm above the lesion epicenter were incubated overnight at 4°C with the primary mouse monoclonal anti-SMI-32 antibody (1:2,500 in PBS-T; 801701, Biolegend, CA, USA). Sections were incubated with AlexaFluor 488 conjugated secondary goat anti-mouse IgG antibody (1:1,000 in PBS-T; Jackson ImmunoResearch, PA, USA) for 3 h at room temperature. Images of LFB and NF-H staining were obtained using a fluorescence microscope (BZ-9000; Keyence Co., Osaka, Japan). The sectional spinal area was determined using LFB-stained images of axial sections from the center of the lesion, captured at × 10 magnification (*n* = 3 in each group). ImageJ software (National Institutes of Health, MD, USA) was used to quantify the LFB- and NF-H-stained sections.

## Results

### Spontaneous Recovery of Locomotion After Contusive Injury

**Figure 2:**
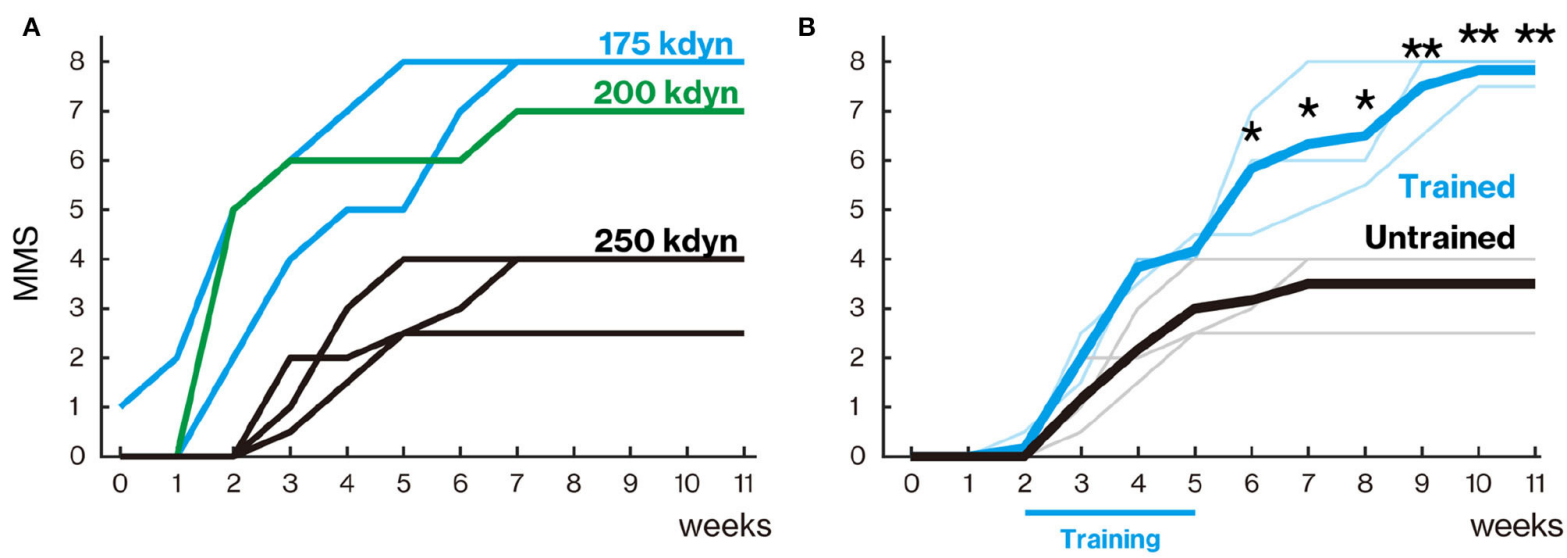
Treadmill training promoted locomotor function recovery. **(A)** Marmoset Motor Scale for Locomotion (MMS) with mild (175 and 200 kdyn) and severe (250 kdyn) contusion groups. Animals with mild contusion gradually recovered their stepping ability, while animals with a severe injury could hardly move their hindlimb until 2 weeks after spinal cord injury (SCI). A detailed explanation of MMS is described in Table 3. **(B)** MMS for the trained and untrained group with severe contusions. Thin and thick lines indicate MMS for each animal and their mean, respectively. Animals in the untrained group gradually recovered and reached a plateau around 5 weeks. In the trained group, the difference in locomotor performance was not seen during the training period (from 2 to 5 weeks after injury); they gradually recovered even after 6 weeks. *p < 0.05; **p < 0.01; Welch's *t*-test.

Locomotor recovery was scored weekly for up to 11 weeks using the MMS (Figure 2A). The MMS and its definitions are presented in Table 3. In the early phase of recovery after a 250-kdyn contusive SCI, marmosets showed no or slightly isolated joint movements. During this phase, movements of hip and knee showed variability: these joints showed contracture in most cases or flaccid hindlimb paralysis in other cases, whereas movements of the ankle showed less variability, and the extent of ankle movement could be readily classified. Consequently, we assessed only ankle movements in the MMS. Ankle movement was classified as slight or extensive based on whether the joint motion is less or more than half of the range of motion. Marmosets with a 250-kdyn contusive SCI showed no or slight ankle joint movements until 2 weeks after injury, and as they recovered, extensive ankle movement was demonstrated. At the end of this early phase, marmosets with a 250-kdyn contusive SCI moved their hip, knee, and ankle extensively and simultaneously.

**Table 3:** Marmoset Motor Scale for Locomotion.

**Score**	**Description**
0	No ankle movement
1	Slight ankle movement
2	Extensive ankle movement
3	Plantar placing of the paw with or without weight support -OR-
	Occasional, frequent or consistent abnormal stepping but no plantar stepping
4	Occasional plantar stepping
5	Frequent or consistent plantar stepping, no coordination -OR-
	Frequent or consistent plantar stepping, some coordination, paws rotated at initial contact and lift off (R/R)
6	Frequent or consistent plantar stepping, some coordination, paws parallel at initial contact (P/R, P/P) -OR-
	Frequent or consistent plantar stepping, mostly coordinated, paws rotated at initial contact and lift off (R/R)
7	Frequent or consistent plantar stepping, mostly coordinated, paws parallel at initial contact (P/R, P/P) -OR-
	Frequent or consistent plantar stepping, mostly coordinated, paws parallel at initial contact and lift off (P/P), and no or occasional toe clearance during forward limb advancement
8	Frequent or consistent plantar stepping, mostly coordinated, paws parallel at initial contact and lift off (P/P), mostly toe clearance during forward limb advancement, and tail down or up and down
9	Frequent or consistent plantar stepping, mostly coordinated, paws parallel at initial contact and lift off (P/P), mostly toe clearance during forward limb advancement, and tail always up

*Slight, Moves less than half of the ankle joint excursion; Extensive, Moves more than half of the ankle joint excursion; Occasional, Stepping less than or equal to half of the time moving; Frequent, Stepping more than half the time moving; Consistent, Plantar stepping almost all of the time moving*.

When the ankle, knee, and hip joint can move well, marmosets can place the plantar surface on the ground, allowing stepping movements. Weight support during plantar placement was scored when the hindquarters were elevated in response to paw placement. Marmosets with a 250-kdyn contusive SCI began to stand 4–6 weeks after injury, but the frequency of plantar stepping was occasional (less than half of the time moving forward).

In the late recovery phase, mildly injured marmosets showed improvements in the finer aspects of locomotion, such as forelimb–hindlimb (FL–HL) coordination, paw position during stance, and toe clearance during swing. These aspects of locomotor recovery were not sustained in marmosets with a 250-kdyn contusive SCI. FL–HL coordination was defined as a one-to-one correspondence between forelimb and hindlimb steps, as in BMS and BBB for rodents. The paw position at the beginning and end of the stance phase, initial contact, and liftoff were determined by assessing whether the middle digits of the hind paw were parallel to the long axis of the body. Tail position during locomotion was rated according to whether it was always down, up and down (held up at least once during locomotion), or up (always held up). The occurrence of any FL–HL coordination was 3–4 weeks after injury in the mild injury group (175 and 200 kdyn; Figure 2A). FL–HL coordination in marmosets as well as in rats preceded the recovery of paw position; on the other hand, coordination and paw position recovered simultaneously in mice.

### Treadmill Training Promoted Significant Locomotor Function Recovery

Animals in the untrained group gradually recovered and reached a plateau at approximately 5 weeks after injury (Figure 2B). In the trained group, the degree of recovery did not change from the untrained group during the training period (from 2 to 5 weeks after injury), but significantly higher recovery was observed from 6 weeks after injury than in the untrained group (6–8 weeks, *p* < 0.05; 9–11 weeks, *p* < 0.01).

### Kinematics Analysis

**Figure 3:**
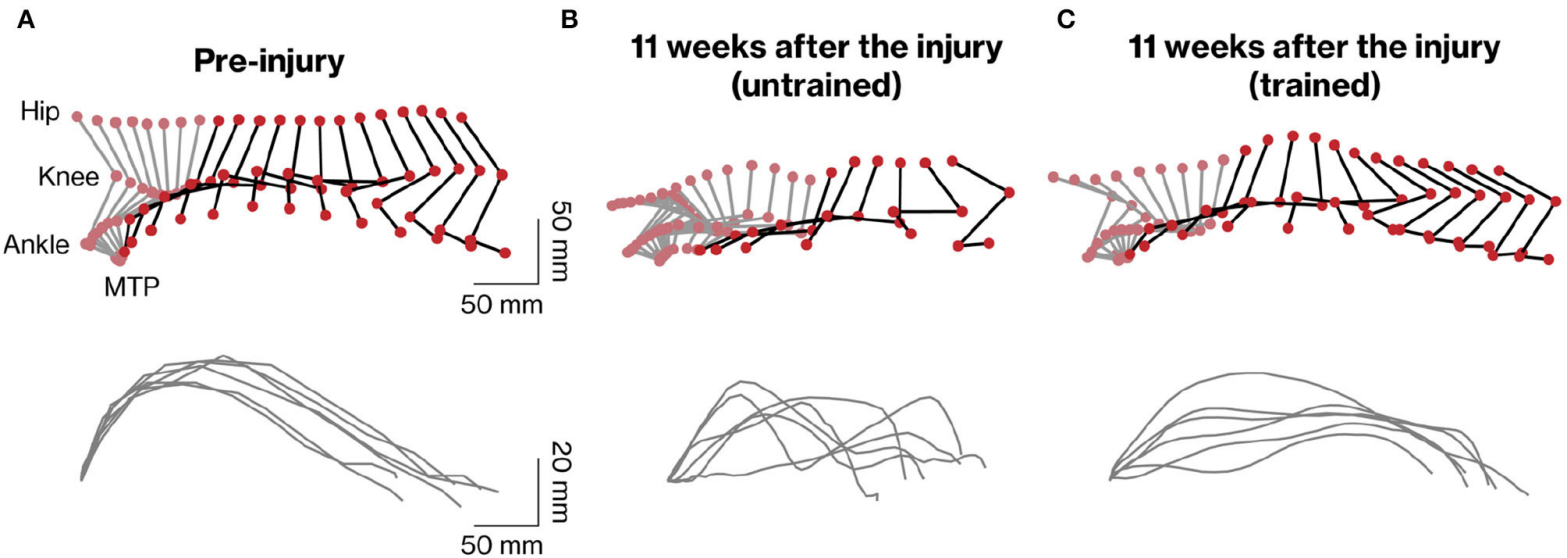
Kinematic analysis of hindlimb movements assessed during quadrupedal walking. Representative stick diagrams of hindlimb movements in pre-injury **(A)** and 11 weeks after injury in untrained **(B)** and trained **(C)** groups are shown (upper panels). Gray and black sticks indicate stance and swing phases, respectively. The time between individual sticks is 35 ms. Successive trajectories of the fifth metatarsal (MTP) are shown for the six consecutive steps in each group (lower panels).

To analyze the stepping ability of marmosets with SCI with and without rehabilitation, the trajectory patterns of the ankles and MTP during stepping were plotted (Figures 3A–C). To clarify the difference in motor function between the groups, the stepping patterns of trained and untrained marmosets were compared at 11 weeks after injury (compare Figures 3B,C). Without rehabilitation, the stepping patterns remained erratic. In contrast, in the trained group, the trajectory before the injury was almost constant.

**Figure 4:**
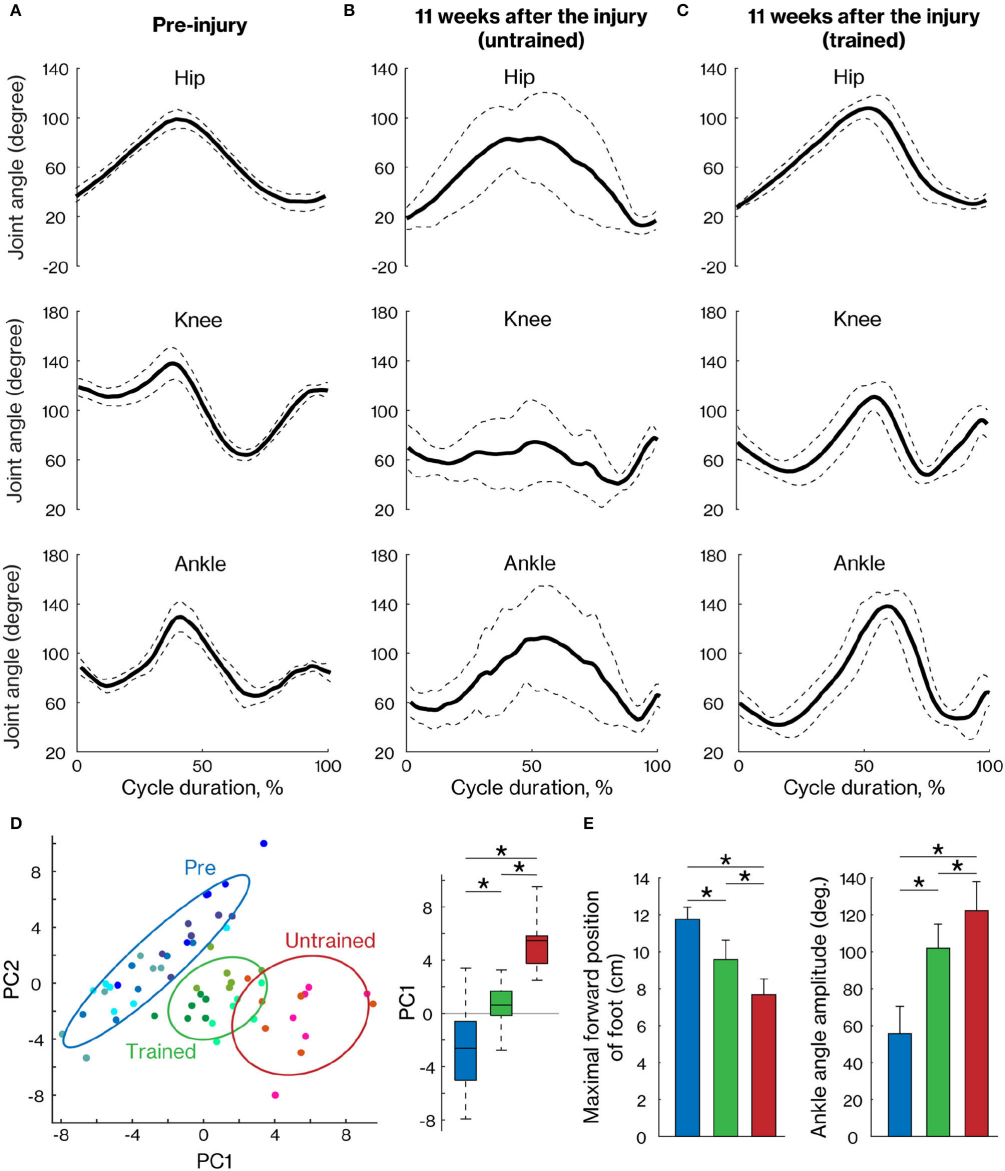
Comparison of the right hindlimb locomotor pattern between trained and untrained groups. The mean (±SD) of angular excursion of the three right hindlimb joints during quadrupedal locomotion in pre-injury **(A)** and 11 weeks after injury in untrained **(B)** and trained groups **(C)**. The solid and dotted lines indicate the mean and SD, respectively. **(D)** The plane was created by PC1 and PC2 (left) and also by the box plots of PC1 scores (right), which were calculated with 47 kinematic parameters. Each plot indicates each cycle data. Least-squares circles help visualize the clusters. Blue, green, and red indicate the pre-training, trained, and untrained groups, respectively. **(E)** Representative kinematic parameters with a high loading factor of PC1. * < 0.05; one-way ANOVA followed by Tukey *post hoc* comparison.

To assess motor recovery at 11 weeks after injury, we measured the angular movement of the three hindlimb joints. The walking pattern displayed by the hindlimb impacted by the contusion was still deficient in untrained marmosets but was well organized in trained marmosets (Figures 4A–C). To comprehensively evaluate the changes, 47 kinematic parameters (Table 2) were extracted in each cycle and PCA was applied. The results show that pre-training, trained and non-trained groups are clustered at different positions on the plane created by PC1 and PC2 (Figure 4D). In particular, PC1 scores, which explain 33% of the variance, were significantly different among the groups, with the trained group closer to the pre-training group than the non-trained group. The maximal forward position of foot and ankle angle amplitude are shown as representative examples of kinematic parameters, which were significantly different between pre-training and trained groups, but are closer than in the nontrained group as well as PC1 scores (Figure 4E).

### Training-Induced Changes in Connectivity Between the Motor Cortex and Lumbar Spinal Cord

**Figure 5:**
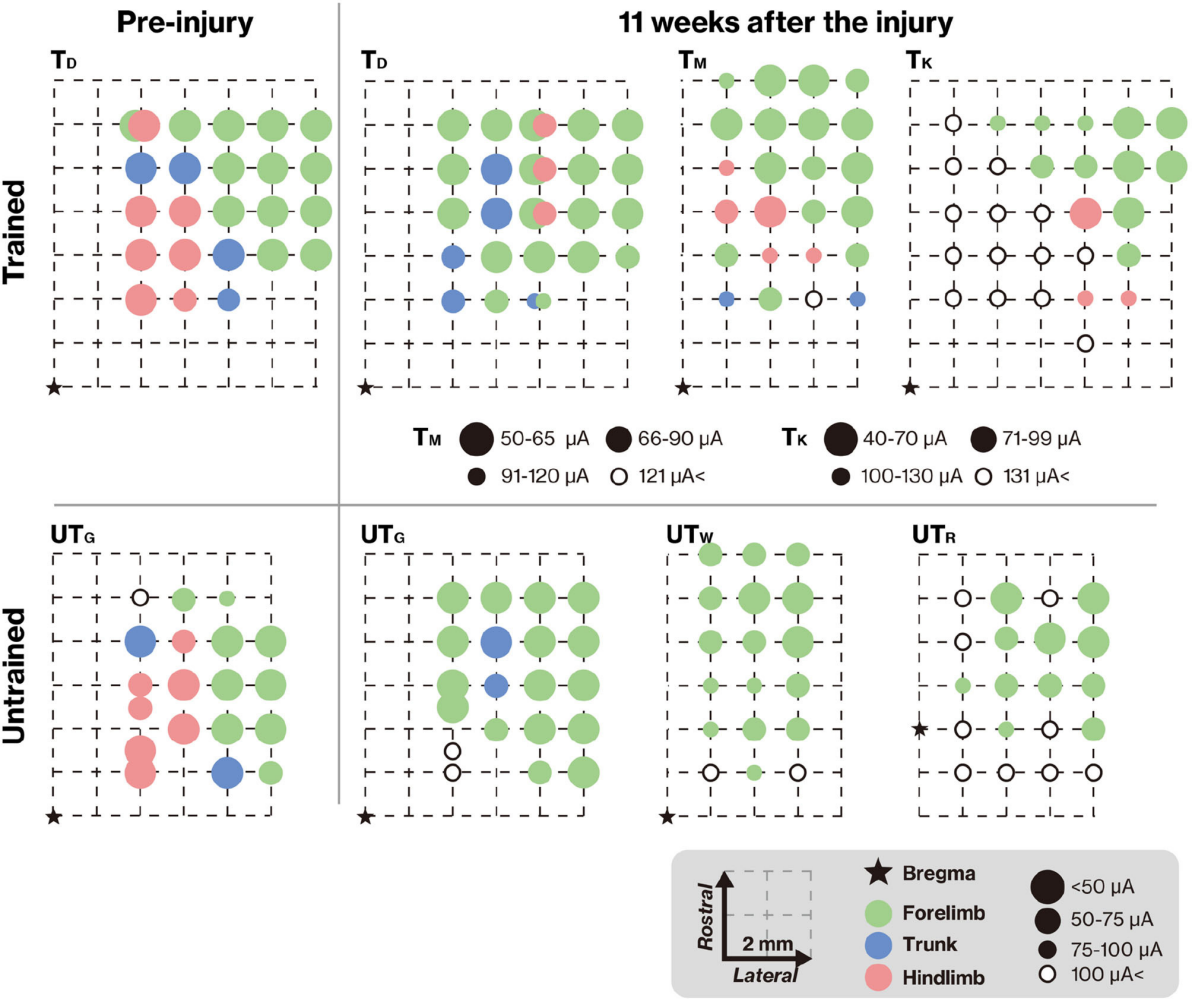
Training-induced changes in the topographic map. The topographic map represents the electrode penetration sites. The corresponding positions of the electrodes are displayed as colors indicating the body territory activated under the lowest intracortical microstimulation (ICMS) current that still causes a movement. The results show that hindlimb movements could be evoked at positions close to the bregma in both T_D_ and UT_G_ (left column). Pre-injury ICMS maps are shown in the right panel. The hindlimb areas in the trained group remained unchanged (upper panel), but after the lesion, the hindlimb areas on the cortical surface in the untrained group were diminished (lower panel). In both groups, the M1 forelimb areas remained after the lesion. Note that different intensities were used for T_M_ and T_K_.

To verify connectivity between the motor cortex and lumbar spinal cord across the lesion, we analyzed the representative hindlimb area of the motor cortex using ICMS. In T_D_ and UT_G_, ICMS was also performed before the lesion (Figure 5, left column) to verify the reorganization of motor maps. The motor maps derived from the ICMS of this study were consistent with those of previous reports (Burish et al., 2008; Burman et al., 2008); the hindlimb motor area was located in the medial part of the motor cortex. Eleven weeks after injury, the hindlimb areas decreased in the untrained group (Figure 5, right panels). In contrast, in the trained group, hindlimb muscle movements were elicited by ICMS. In the T_M_, the emerged hindlimb area seems to be the original hindlimb area; however, in T_D_ and T_K_, hindlimb muscle movements were not elicited in original hindlimb areas and were elicited in ectopic areas (i.e., in the original forelimb area). This indicates that training promotes reorganization of the connection between newly emerged hindlimb areas and the lumbar spinal cord.

### Histological Analysis

**Figure 6:**
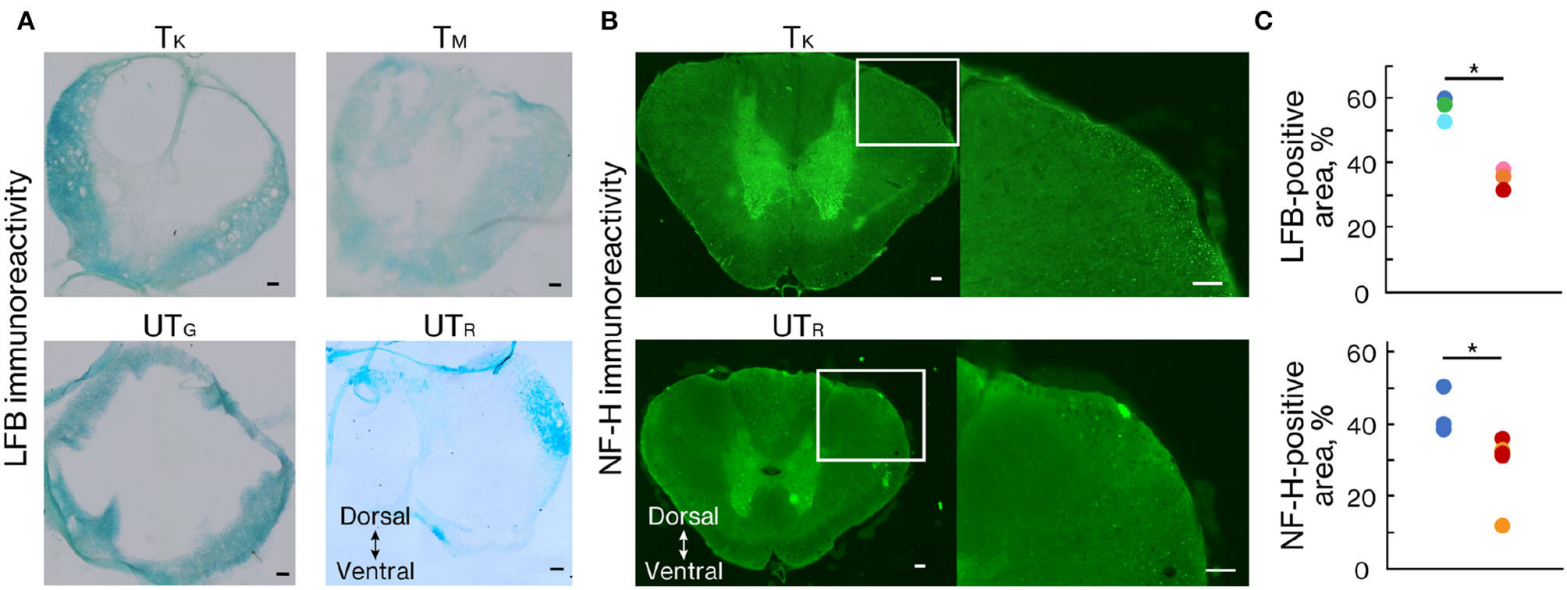
Rehabilitation enhanced myelinated areas and fibers. **(A)** Luxol fast blue (LFB) and **(B)** NF-H staining 11 weeks after injury. **(C)** Percentage of the positive area in trained (*n* = 3) and untrained (*n* = 3) groups. The LFB-positive area was significantly more prominent at the lesion epicenter in the trained group than in the untrained group. The NF-H-positive area was significantly larger in the trained group than in the untrained group. Conventions are the same as in Figure 4D. *p < 0.05; Welch's *t*-test. Scale bars = 100 μm.

To demonstrate the effects of rehabilitation on the prevention of demyelination or the promotion of remyelination after SCI, LFB staining was performed 11 weeks after injury (Figure 6A). LFB-positive areas within the lesion epicenter were significantly larger in the trained group than in the untrained group (Figures 6A,C; *p* < 0.01). We also performed an immunohistochemical analysis of NF-H to quantify the neuronal fibers passing through the lesion related to locomotor recovery. The NF-H-positive areas were comparable between the two groups in animals with chronically injured spinal cords at all tested levels (Figures 6B,C; *p* < 0.05).

## Discussion

In this study, we developed a contusive SCI model with therapeutic intervention for locomotor rehabilitation in nonhuman primates. ICMS elicited hindlimb movement in the trained group, and significant locomotor recovery was detected on both the original open-field scale and kinematics analysis. Histological analysis showed that marmosets in the training group had less tissue damage than those in the non-training group, suggesting that there might be a neuroprotective effect.

Rehabilitation regained the connection between supraspinal input and the lumbar spinal cord. Previous studies have shown that rehabilitative training causes the secretion of neurotrophic factors, such as brain-derived neurotrophic factors (BDNF; Fouad and Tetzlaff, 2012). BDNF is involved in neuroprotection, axonal sprouting, and regeneration (Kafitz et al., 1999; Vavrek et al., 2006). Although the present study did not assess these factors because of the small number of samples, our histological findings with larger myelinated and axonal areas in the trained group indicate that these trophic factors may be upregulated and promote neural protection.

As the recovery of locomotor functions depends on the preservation of supraspinal input (Cote et al., 2017), the degree of connection between supraspinal input and the lumbar spinal cord is also a possible mechanism for recovery in this study. Most clinical studies examining the influence of the supraspinal tract have been performed using transcranial magnetic stimulation of the motor cortex (Kamida et al., 1998; Friedli et al., 2015), and ICMS (Alstermark et al., 2004; Schmidlin et al., 2004), leading to preferential activation of the CST (Yang and Gorassini, 2006). In this study, the effect of supraspinal input was tested by ICMS, and the muscle activity of the lower limbs was induced in the training group (Figure 5). To date, there is no evidence to address the changes in the functional map induced in the hindlimb representation of the motor cortex in primates. In rodent models, it is generally accepted that the expansion of motor maps is related to functional recovery (Fouad and Tetzlaff, 2012) and can be facilitated by rehabilitative training (Girgis et al., 2007; Ishida et al., 2016). The main reason for this restoration may be increased collateral sprouting of CST fibers in parallel with cortical map changes (Fouad and Tetzlaff, 2012). Because primate species have greater potential for CST regeneration than rodents do (Friedli et al., 2015), the cortical map changes that occurred in trained marmosets in our study may have been caused by CST plasticity, which in turn may have contributed to motor functional improvement. However, our results showed that the restoration of corticospinal connectivity did not directly indicate the sprouting and regeneration of a CST. For example, a study of rehabilitation after CST injury in rats showed that muscle movements were elicited by ICMS, *via* the cortico-rubral tract (Ishida et al., 2016). To validate CST plasticity, it is necessary to visualize CST using neural tracers.

### A Severe Contusion Model With Rehabilitation in Marmosets

Nonhuman primates may provide many advantages in investigating the safety and efficacy of various therapies to promote functional recovery after SCI. In the field of rehabilitative areas, several primate studies have examined the effectiveness of using incomplete transection models to test the effects of a treatment that promotes sprouting from spared axons and, perhaps, axon regeneration (Cote et al., 2017). However, these transection models do not resemble human SCI and are therefore not clinically relevant (Courtine et al., 2007; Kwon et al., 2011). Hence, it is important to establish rehabilitation methods in primate contusion models to obtain clinically applicable knowledge.

Furthermore, a few contusive SCI studies have been carried out in large primates because they are difficult to handle, labor-intensive, and require indispensable skilled daily care (Babu and Namasivayam, 2008). In addition, weight-supported treadmill rehabilitation requires an extremely hard step of putting a weight support jacket on monkeys and some other special rehabilitative setups (Krucoff et al., 2017). On the other hand, small marmosets are easy to handle, care for, and rehabilitate (Okano, 2021). This advantage over the use of macaques enabled the development of a rehabilitation model for SCI.

In general, the recovery effect is limited if rehabilitation therapy is applied alone, and its efficacy depends on the severity of the injury. Patients with motor incomplete SCI (AIS C or D) showed some improvement in overground walking (Cote et al., 2017), while patients with more severe injury (AIS A and B) showed increased electromyographic amplitude and improved mutual activation of the extensor and flexor muscles, but no recovery of independent stepping ability after rehabilitation (Dietz et al., 2002; Forrest et al., 2008). These studies suggest that functional recovery after a severe SCI requires a combination of rehabilitation and biological repair strategies, such as pharmacological (Ito et al., 2018) and stem cell therapies (Shinozaki et al., 2021; Tashiro et al., 2021).

## Data Availability Statement

The raw data supporting the conclusions of this article will be made available by the authors, without undue reservation.

## Ethics Statement

The animal study was reviewed and approved by Animal Research Committee of Keio University School of Medicine.

## Author Contributions

All authors listed have made a substantial, direct, and intellectual contribution to the work and approved it for publication.

## Funding

This research was supported by General Insurance Association of Japan Medical Research Grants 2021 and by AMED under grant nos. JP21bm0204001h, JSPS KAKENHI, JP19H03983, and JP20H05480.

## Conflict of Interest

JU is the founder and Representative Director of the University Startup Company, Connect, Inc., for the research, development, and sale of rehabilitation devices, including the brain–computer interface. He received a salary from Connect, Inc., and held shares in Connect, Inc. This company has no relationship with the present study. HO is a compensated scientific consultant of San Bio Co., Ltd., and K Pharma, Inc., and has received research funding from Dainippon Sumitomo Pharmaceutical Co., Ltd. MN is a compensated scientific consultant at K Pharma, Inc. YS and TK are founders of ALAN, Inc., and hold shares in ALAN, Inc. The remaining authors declare that the research was conducted in the absence of any commercial or financial relationships that could be construed as a potential conflict of interest.

## Publisher's Note

All claims expressed in this article are solely those of the authors and do not necessarily represent those of their affiliated organizations, or those of the publisher, the editors and the reviewers. Any product that may be evaluated in this article, or claim that may be made by its manufacturer, is not guaranteed or endorsed by the publisher.
